# Influence of inflammatory conditions provided by macrophages on osteogenic ability of mesenchymal stem cells

**DOI:** 10.1186/s13287-020-1578-1

**Published:** 2020-02-13

**Authors:** Gema Vallés, Fátima Bensiamar, Leila Maestro-Paramio, Eduardo García-Rey, Nuria Vilaboa, Laura Saldaña

**Affiliations:** 1 Hospital Universitario La Paz. IdiPAZ, Paseo de la Castellana 261, 28046 Madrid, Spain; 2 Centro de Investigación Biomédica en Red de Bioingeniería, Biomateriales y Nanomedicina, CIBER-BBN, Madrid, Spain; 3 Departamento de Cirugía Ortopédica y Traumatología, Hospital Universitario La Paz-IdiPAZ, Madrid, Spain

**Keywords:** Mesenchymal stem cells, Osteogenesis, Migration, Attachment, Macrophage polarization, Cytokines

## Abstract

**Background:**

The mechanisms by which macrophage phenotype contributes to mesenchymal stem cells (MSC)-mediated bone repair remain unclear. In this work, we investigated the influence of factors released by human macrophages polarized to a pro-inflammatory or an anti-inflammatory phenotype on the ability of human MSC to attach, migrate, and differentiate toward the osteoblastic lineage. We focused on the role of TNF-α and IL-10, key pro-inflammatory and anti-inflammatory cytokines, respectively, in regulating MSC functions.

**Methods:**

MSC were treated with media conditioned by pro-inflammatory or anti-inflammatory macrophages to study their influence in cell attachment, migration, and osteogenic differentiation. The involvement of TNF-α and IL-10 in the regulation of MSC functions was investigated using neutralizing antibodies and recombinant cytokines.

**Results:**

Treatment of MSC with media conditioned by pro-inflammatory or anti-inflammatory macrophages promoted cell elongation and enhanced MSC ability to attach and migrate. These effects were more noticeable when MSC were treated with media from pro-inflammatory macrophages. Interestingly, MSC osteogenic activity was enhanced by factors released by anti-inflammatory macrophages, but not by pro-inflammatory macrophages. Significant IL-10 levels originated from anti-inflammatory macrophages enhanced MSC osteogenesis by increasing ALP activity and mineralization in MSC layers cultured under osteogenic conditions. Moreover, macrophage-derived IL-10 regulated the expression of the osteogenic markers *RUNX2*, *COL1A1*, and *ALPL*. Notably, low TNF-α levels secreted by anti-inflammatory macrophages increased ALP activity in differentiating MSC whereas high TNF-α levels produced by pro-inflammatory macrophages had no effects on osteogenesis. Experiments in which MSC were treated with cytokines revealed that IL-10 was more effective in promoting matrix maturation and mineralization than TNF-α.

**Conclusions:**

Factors secreted by pro-inflammatory macrophages substantially increased MSC attachment and migration whereas those released by anti-inflammatory macrophages enhanced MSC osteogenic activity as well as cell migration. IL-10 was identified as an important cytokine secreted by anti-inflammatory macrophages that potentiates MSC osteogenesis. Our findings provide novel insights into how environments provided by macrophages regulate MSC osteogenesis, which may be helpful to develop strategies to enhance bone regeneration.

## Introduction

Inflammatory response mediated by macrophages plays a vital role in fracture healing [1, 2]. Upon bone injury, monocytes are recruited to the wound site and differentiate into activated macrophages, a major source of cytokines involved in bone repair and regeneration. Macrophage populations can adopt differential functional profiles ranging from the pro-inflammatory polarization state M1 to the anti-inflammatory M2. In the initial phase of inflammation, M1-type macrophages secrete high amounts of pro-inflammatory cytokines such as tumor necrosis factor-alpha (TNF-α). TNF-α, a primary mediator in the inflammatory reaction, promotes the recruitment of inflammatory and stromal cells, stimulates angiogenesis and is essential for bone fracture repair, as demonstrated in TNF-α receptor-deficient mice [3, 4]. In the later stages of inflammation, M2-type macrophages secrete anti-inflammatory cytokines and growth factors, which mediate the resolution of inflammation and tissue repair. Among them, interleukin-10 (IL-10) is a key anti-inflammatory cytokine in the bone regenerative process. IL-10-deficient mice display osteopenia, mechanical fragility of bones, and decreased bone formation [5]. The outcome of bone repair is tightly regulated by the inflammatory milieu, leading the imbalance between pro-inflammatory and anti-inflammatory cytokines during bone healing to chronic inflammation, aberrant bone repair, or excessive ossification.

The early step of bone formation involves the migration of mesenchymal stem cells (MSC), which undergo proliferation and differentiation along the osteoblastic lineage. Indeed, MSC implantation has emerged as a promising strategy for promoting bone regeneration in several clinical conditions, including delayed unions and non-unions, critical-sized bone defects, periprosthetic osteolysis, osteonecrosis, and inflammatory bone disorders [6–10]. The benefits of MSC therapy are mainly attributed to paracrine effects via soluble factors, exerting both immunoregulatory and regenerative actions [11]. Although implanted MSC may act as osteoprogenitors [12–14], most evidence indicates that newly formed bone is mainly derived from host progenitors. The mechanisms by which macrophages contribute to MSC-mediated tissue repair remain unclear. TNF-α, which is highly expressed in macrophages during the early inflammatory phase after fracture [15], promotes MSC migration and plays an important role in the activation of MSC immunoregulatory properties [16–18]. Moreover, pro-inflammatory cytokines can directly impact MSC commitment to the osteoblastic lineage. Thus, TNF-α contained in supernatants from human fractured tibial bone enhances MSC osteogenic differentiation in vitro [19]. Further studies using recombinant TNF-α show that its effect on MSC osteogenesis depends on the dose and the length of treatment [19–23]. Regarding anti-inflammatory cytokines, their effects on bone healing have been largely explained by their ability to inhibit the production of pro-inflammatory factors by macrophages [24]. However, very little attention has focused on their direct effects on MSC. We recently reported that IL-10 originated from pro-resolving macrophages enhances MSC immunomodulatory potential in vitro [18]. In addition, a recent study showed that physiological doses of recombinant IL-10 stimulate MSC osteogenic differentiation while higher pathological doses inhibit it [25].

In the present study, we investigated the influence of factors produced by human macrophages polarized to a pro-inflammatory or an anti-inflammatory phenotype on the ability of MSC to attach, migrate, and differentiate toward the osteoblastic lineage. We focused on the role of TNF-α and IL-10, key pro-inflammatory and anti-inflammatory cytokines, respectively, in regulating MSC functions.

## Methods

### Isolation and culture of primary human macrophages

**Fig. 1:**
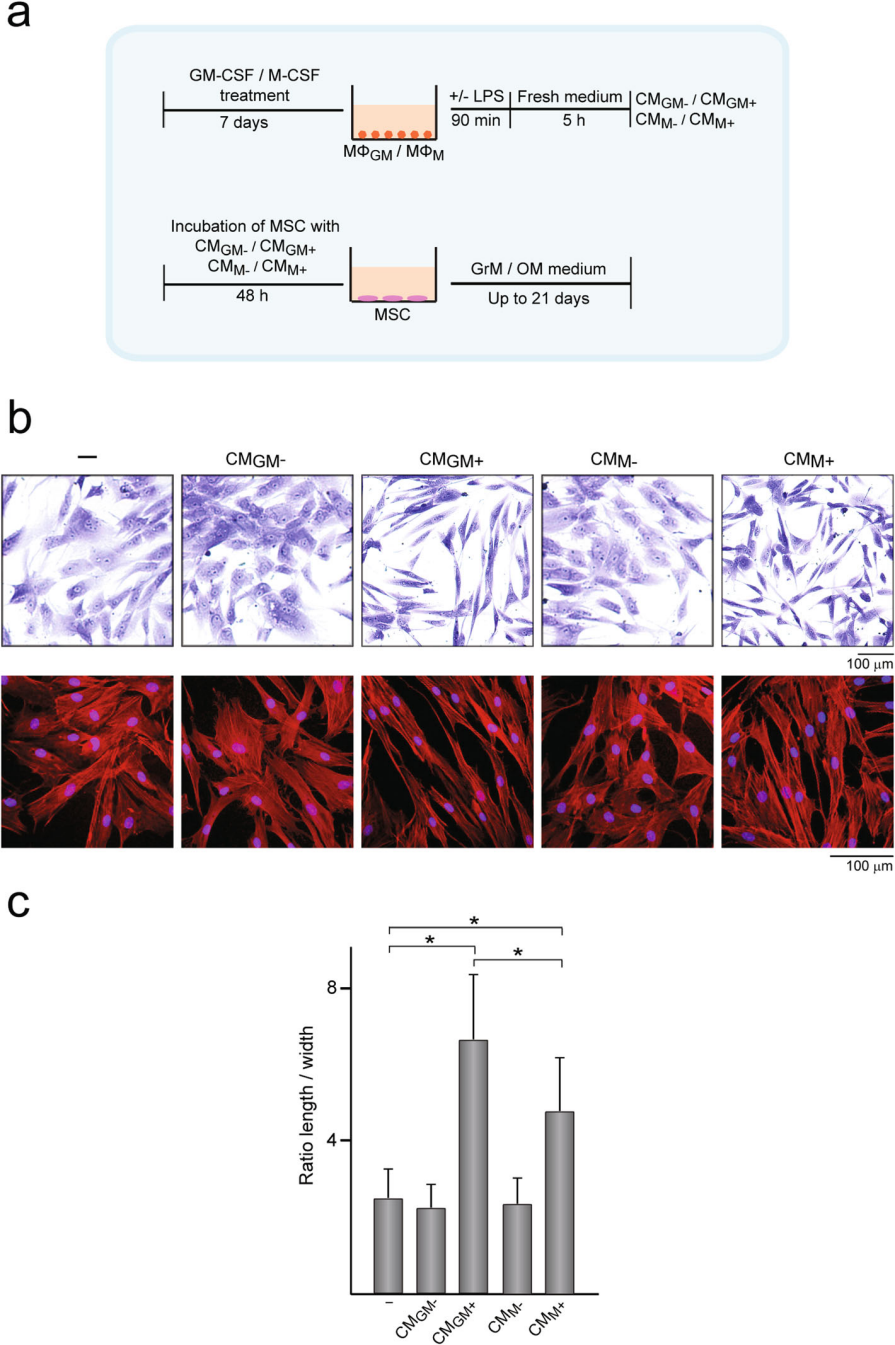
Morphology of MSC treated with CM. **a** Experimental scheme used for generating conditioned medium (CM) from macrophages and for MSC treatments. CM_GM+_ and CM_M+_ were obtained from MΦ_GM_ and MΦ_M,_ respectively, that were treated with LPS for 90 min, thoroughly washed with PBS, and further incubated in fresh medium for 5 h. CM_GM−_ and CM_M−_ were obtained from MΦ_GM_ and MΦ_M_, respectively, that were not treated with LPS but subjected to identical experimental manipulations. MSC were incubated or not with CM for 48 h, thoroughly washed with PBS, and further incubated in growth (GrM) or osteogenic (OM) medium up to 21 days. **b** Optical microscope images showing the morphology of MSC treated or not (−) with CM after crystal violet staining (upper row). Confocal microscope images (maximum projection) showing actin (red) and nuclei (blue) in MSC treated or not with CM (lower row). **c** Ratio of cell length to width as measure of cell elongation. *p < 0.05 between the indicated conditions

Buffy coats were obtained from 15 healthy blood donors anonymously provided by the Comunidad de Madrid Blood Bank (Madrid, Spain). This study was approved by the Human Research Committee of Hospital Universitario La Paz (date of approval 03/06/2015). All experiments were carried out in accordance with the approved guidelines and regulations. Human peripheral blood mononuclear cells (PBMC) were isolated from buffy coats by Ficoll-Paque Plus (GE Healthcare Bio-sciences, Uppsala, Sweden) density gradient centrifugation. PBMC were seeded at a density of 15 × 10^6^/well in 6-well plates and allowed to adhere for 1 h in serum-free RPMI (Lonza, Basel, Switzerland). Attached cells were incubated for 7 days in RPMI supplemented with 10% (v/v) heat-inactivated fetal bovine serum (FBS) and 200 U/ml granulocyte macrophage colony-stimulating factor (GM-CSF) or 20 ng/ml macrophage colony-stimulating factor (M-CSF) (both from Peprotech, London, UK). Cytokines were added every 2 days. Macrophages generated after incubation with GM-CSF (MΦ_GM_) expressed the M1 markers CD80 and CCR7 while they were devoid of the cell-surface receptor CD163, a marker of M2 macrophages. In contrast, macrophages generated after incubation with M-CSF (MΦ_M_) expressed high levels of CD163 and very low levels of CD80 and CCR7 [18]. Conditioned medium (CM) was obtained from MΦ_GM_ and MΦ_M_ treated (CM_GM+_ and CM_M+_, respectively) or not (CM_GM-_ and CM_M-_, respectively) with 10 ng/ml lipopolysaccharide (LPS) (Sigma, Madrid, Spain) for 90 min, washed three times with phosphate-buffered saline (PBS) and cultured for 5 h in RPMI supplemented with 10% FBS in the absence of LPS. The experimental scheme used to generate CM is shown in Fig. 1a. CM was clarified by centrifugation at 1200 g for 10 min. Levels of TNF-α, IL-6, IL-1β, IL-10, IL-8, and monocyte chemoattractant protein-1 (MCP-1) in CM were determined using BD CBA Flex Sets (BD Biosciences, San Jose, CA, USA). The data were acquired using a FACSCalibur flow cytometer and analyzed with the FCAP Array Software version 3.0 (BD Biosciences). The detection limits were 3.7 pg/ml for TNF-α, 2.5 pg/ml for IL-6, 2.6 pg/ml for IL-1β, 3.3 pg/ml for IL-10, 1.2 pg/ml for IL-8, and 1.3 pg/ml for MCP-1.

### MSC culture and treatments

Human MSC derived from bone marrow were purchased from Lonza and expanded in a defined growth medium consisting of basal medium and supplements (Lonza). All experiments were performed below the seventh cell passage. Experiments were performed in duplicate using MSC isolated from five different donors aged 18–31 years. MSC were incubated for 48 h in DMEM supplemented with 15% (v/v) heat-inactivated FBS (growth medium) or in a mixture of equal volumes of growth medium and CM. When indicated, and prior to addition to MSC, CM was incubated for 1 h at 37 °C with 1 μg/ml neutralizing antibody against TNF-α or IL-10 (Biolegend, San Diego, CA, USA). In some experiments, MSC were incubated in growth medium containing human TNF-α or IL-10 at 0.1, 1, or 10 ng/ml (Peprotech). This concentration range was selected according to TNF-α and IL-10 levels in CM_GM+_ or CM_M+_. In differentiation assays, MSC treated with CM or cytokines were washed three times with PBS and incubated in osteogenic medium consisting of growth medium supplemented with 3 × 10^−4^ M ascorbic acid, 10^−2^ M β-glycerophosphate, and 10^−7^ M of dexamethasone (all from Sigma) for 3–21 days. As controls, untreated cells were incubated in the growth medium. The experimental scheme is shown in Fig. 1a.

### Cell morphology, attachment, and migration

To analyze cell morphology, 2 × 10^4^ MSC were seeded in 8-well chamber slides and incubated for 48 h in 200 μl of a mixture of equal volumes of growth medium and CM or in 200 μl of growth medium supplemented or not with the indicated cytokines. Cells were washed with PBS, stained with crystal violet, and observed under a phase-contrast microscope (Nikon Diaphot, Tokio, Japan). Cell elongation was determined by analyzing a total of 100 cells per condition, randomly selected from 6 representative images obtained from 3 independent experiments with similar results. Cells were manually outlined using ImageJ v1.34 image analysis software (http://rsbweb.nih.gov/ij), which fits each cell to the fittest ellipse. The lengths of the major and the minor axis of the fittest ellipse correspond to cell length and width, respectively. Elongation was defined as the ratio of the major axis to the minor axis. Parallel sets of cells were fixed with 4% (w/v) formaldehyde in PBS, permeabilized with 0.1% Triton X-100 in PBS and double-stained with PBS containing 4 × 10^−7^ M phalloidine-tetramethylrhodamine (TRITC) (Sigma) and 3 × 10^−6^ M 4,6-diamidino-2-phenylindole (DAPI, Sigma). Images of fluorescently stained cells were obtained using a Leica TCS SPE confocal microscope (Wetzlar, Germany).

For cell attachment assays, 10^6^ MSC were seeded in 100 mm diameter culture dishes and incubated for 48 h in 10 ml of a mixture of equal volumes of growth medium and CM or in 10 ml of growth medium supplemented or not with the indicated cytokines. Cells were detached and seeded at a density of 10^5^/well in 24-well plates for 0.5, 1, 2, or 3 h in growth medium. After washing extensively with PBS, attached cells were trypsinized and the number of live cells at each time point was quantified by the trypan blue dye exclusion method. To determine cell migration, 2 × 10^4^ MSC were seeded on tissue culture plastic surfaces confined within squared silicone barriers (Flexiperm, Sigma) placed in 6-well plates and incubated for 48 h in 200 μl of a mixture of equal volumes of growth medium and CM or in 200 μl of growth medium. After washing three times with PBS, the silicon barrier was removed, allowing cells to migrate, and cultures were incubated for 5 days in growth medium. Cells were subsequently stained with crystal violet and observed under a phase-contrast microscope. In some experiments, MSC were incubated in growth medium supplemented with human TNF-α or IL-10 at 1 ng/ml or with IL-10 at 10 ng/ml and cell attachment and migration were determined as described above.

### Cell viability

10^5^ MSC were seeded in 12-well plates and incubated for 48 h in 1 ml of a mixture of equal volumes of growth medium and CM or in 1 ml of growth medium supplemented or not with the indicated cytokines. After washing three times with PBS, MSC were incubated for 1, 3, and 7 days in growth medium. Cell viability was determined using the alamarBlue assay (Biosource, Nivelles, Belgium). Briefly, MSC were incubated in DMEM containing 10% alamarBlue dye for 3 h and the fluorescence emitted by cell-reduced alamarBlue was quantified using a spectrofluorimeter (Synergy4, Evry, France).

### ALP activity and cell layer calcification assays

2 × 10^5^ MSC were seeded in 6-well plates and incubated for 48 h in 2 ml of a mixture of equal volumes of growth medium and CM or in 2 ml of growth medium supplemented or not with the indicated cytokines. After washing with PBS, MSC were induced to undergo differentiation by incubation in osteogenic medium up to 21 days. As controls, untreated cells were incubated in growth medium. Media were partially replaced every 3 days. After 14 days of culture, cell layers were extracted with 5 × 10^−1^ M NaCl, 5 × 10^−2^ M Tris-HCl pH 8.0, and 1% Triton X-100 and supplemented with a mixture of protease inhibitors. Alkaline phosphatase (ALP) activity was assessed in cell layers by determining the release of p-nitrophenol from p-nitrophenyl phosphate (Sigma). The data were normalized to the total protein contents determined by a Bradford-based protein assay (Bio-Rad Laboratories Inc., Hercules, CA). The degree of cell layer calcification was assessed in cells cultured for 3–21 days using alizarin red staining. Briefly, cells were fixed with ethanol and stained with 40 mM alizarin red S in deionized water at pH 4.2. Images of stained cell layers were obtained using a phase-contrast microscope. The bound stain was eluted with 10% (w/v) cetylpyridinium chloride and the absorbance at 562 nm was measured using a spectrofluorometer.

### Gene expression

2 × 10^5^ MSC were seeded on 6-well plates and incubated for 48 h in 2 ml of a mixture of equal volumes of growth medium and CM or in 2 ml of growth medium supplemented or not with the indicated cytokines. MSC were harvested immediately after treatment with CM or cytokines or after washing with PBS and further incubation in osteogenic medium for 3–21 days. Total RNA was isolated using TRI Reagent (Molecular Research Center, Inc., Cincinnati, OH, USA). Complementary DNAs were prepared from total RNA using the Transcriptor Reverse Transcriptase and an anchored-oligo (dT)_18_ primer (Roche Applied Science, Indianapolis, IN, USA). Real-time quantitative PCR was performed using the LightCycler FastStart DNA Master SYBR Green I and a LightCycler instrument (Roche). Quantitative expression values were extrapolated from standard curves and normalized to the expression values of glyceraldehyde 3-phosphate dehydrogenase (*GAPDH*). Due to a lack of stability of *GAPDH* expression in cells incubated under osteogenic conditions, quantitative expression values from cells cultured in osteogenic medium were normalized to the mean of the expression values of hypoxanthine-guanine phosphoribosyltransferase (*HPRT1*) and beta-glucuronidase (*GUSB*). Specific oligonucleotide primers were runt-related transcription factor 2 (*RUNX2)*, 5′-ATGATGACACTGCCACCTCTGA-3′ (forward primer, F), 5′-GGCTGGATAGTGCATTCGTG-3′, (reverse primer, R); collagen type I alpha 1 *(COL1A1)*, 5′-CGGGCCTCAAGGTATTGCT-3′ (F) and 5′-GGGACCTTGTTTGCCAGGTT-3′ (R); *ALPL*, 5′- GACTAAGAAGCCCTTCACTGCCAT-3′ (F), 5′- GACTGCGCCTGGTAGTTGTT-3′ (R); *GAPDH*, 5′-GTGAAGGTCGGAGTCAACG-3′ (F), 5′-GAAGATGGTGATGGGATTTCC-3′(R); *HPRT1*, 5′-ACCCCACGAAGTGTTGGATA-3′ (F), 5′-AAGCAGATGGCCACAGAACT-3′(R) and *GUSB*, 5′-AAACGATTGCAGGGTTTCAC-3′ (F), 5′-CTCTCGTCGGTGACTGTTCA-3′(R).

### Statistical analysis

The statistical analyses were performed using the Statistical Program for Social Sciences version 11.5 (SPSS Inc., Chicago, IL, USA). Experiments were carried out in duplicate and data are presented as means ± SD of at least five independent experiments. Shapiro-Wilk and Kolmogorov-Smirnov normality tests were used to evaluate whether the data followed normal distribution. Quantitative data were tested using one-way analysis of variance (ANOVA) followed by Bonferroni’s multiple comparison test or Kruskal-Wallis followed by Dunn’s multiple comparison test, depending on whether the data were parametric or nonparametric, respectively. The level of significance was set to *p* < 0.05.

## Results

### Inflammatory factors secreted by macrophages affect MSC morphology, adhesion, and migration

**Fig. 2:**
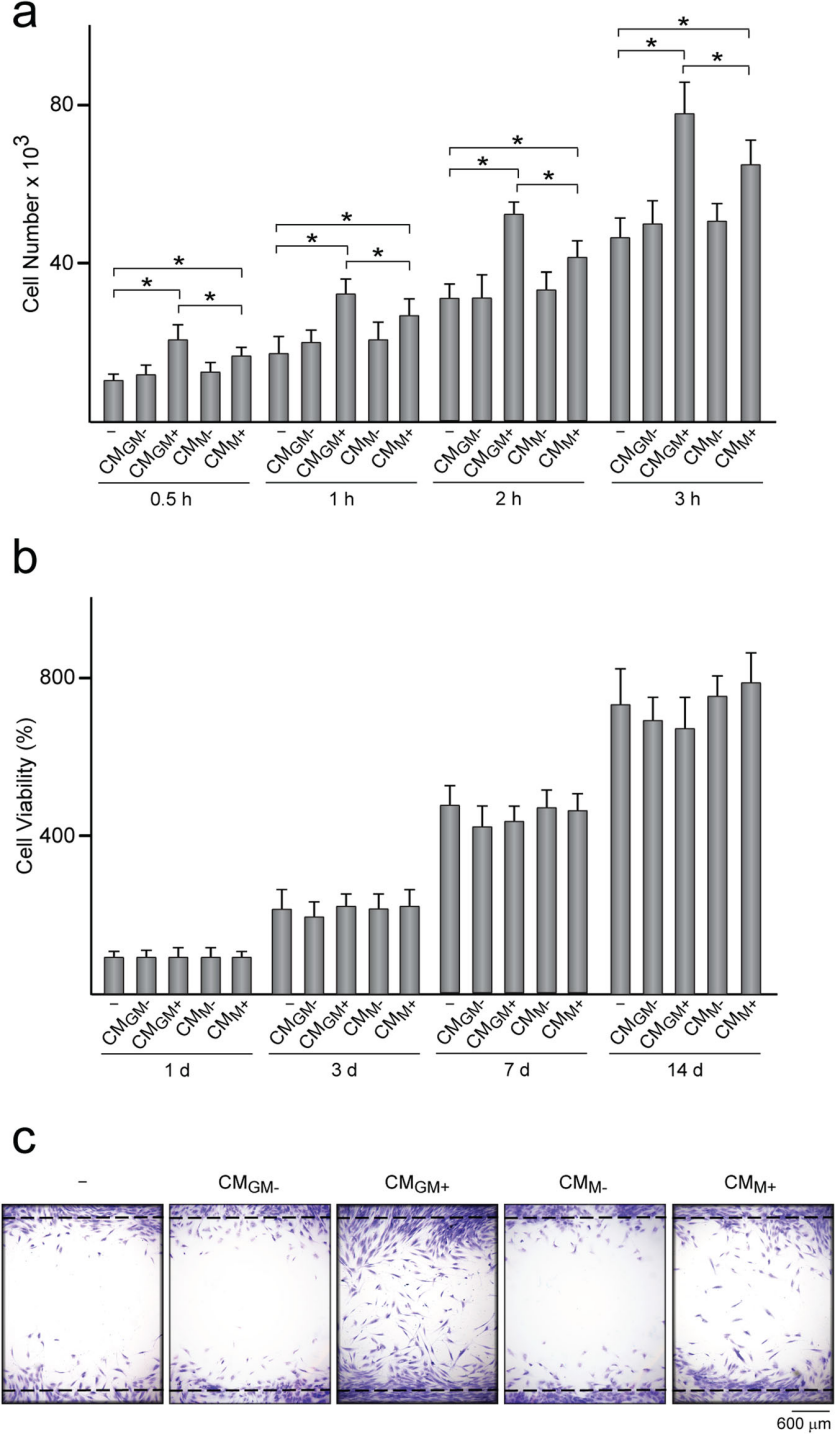
Adhesion, viability and migration of MSC treated with CM. **a** Number of MSC treated or not (−) with CM from MΦ_GM_ and MΦ_M_ activated (CM_GM+_ and CM_M+_, respectively) or not (CM_GM−_ and CM_M−_ , respectively) with LPS and further incubated in growth media (GrM) for the indicated time points. **b** Viability of MSC treated or not with CM and further incubated in GrM for the indicated time points. The data are relative to those measured in each group at day 1, which were given an arbitrary value of 100. **c** Migration capacity of MSC seeded inside the area confined by squared silicone barriers, treated or not with CM and allowed to migrate for 5 days. Optical microscope images of cells stained with crystal violet. *p < 0.05 between the indicated conditions

MSC were treated with CM from MΦ_GM_ or MΦ_M_ activated or not with LPS to examine the effects of inflammatory cytokines on MSC (Fig. 1a). First, we determined the concentrations in CM of some inflammatory factors whose levels are known to change during the inflammatory response subsequent to bone injury. CM_GM+_ contained higher levels of the classical pro-inflammatory cytokines TNF-α, IL-6, and IL-1β and lower levels of the anti-inflammatory cytokine IL-10 than CM_M+_ (Table 1). Moreover, the levels of the inflammatory chemokines IL-8 and MCP-1 were higher in CM_GM+_ than in CM_M+_. In contrast, IL-10 and IL-1β levels could not be detected in CM from non-activated MΦ_GM_ or MΦ_M_, which contained low concentrations of TNF-α, IL-6, IL-8, and MCP-1 (Table 1). MSC treated for 48 h with CM from activated macrophages displayed a much more elongated morphology than untreated MSC (Fig. 1b, c). Cell elongation was more pronounced after treatment with CM_GM+_ than with CM_M+_ (Fig. 1c). The organization of actin cytoskeleton, involved in the acquisition of cell shape, showed differences in untreated and treated MSC. Actin bundles of MSC treated with CM_GM+_ or CM_M+_ organized in closely packed parallel arrays throughout the stretched cell body whereas actin filaments of untreated MSC were more loosely spaced (Fig. 1b). No differences in cell shape and elongation or in actin arrangement were found between MSC treated with CM from non-activated macrophages and untreated cells (Fig. 1b, c). To assess whether exposure to CM affects MSC attachment, cells were seeded on tissue culture plastic and subsequent cultured up to 3 h. Attachment of MSC untreated and treated with CM from non-activated macrophages was similar (Fig. 2a). In contrast, the treatment of MSC with CM from activated macrophages increased cell attachment at all evaluated time points. The number of attached cells was higher when MSC were treated with CM_GM+_ than with CM_M+_. Viability of MSC incubated in growth medium up to 14 days was unaffected by CM treatment (Fig. 2b) Next, we determined whether factors secreted from macrophages regulate MSC migratory activity. Treatment with CM from activated macrophages increased MSC migration, being this effect higher in MSC treated with CM_GM+_ than with CM_M+_ (Fig. 2c). No effect on migratory activity was observed in cells treated with CM from non-activated macrophages. Together, these data indicate that CM from activated macrophages, which contains high levels of inflammatory mediators, promotes MSC elongation, and enhances MSC ability to attach and migrate. These effects were more noticeable when MSC were treated with CM_GM+_ than with CM_M+_.

**Table 1:** Levels of inflammatory cytokines in conditioned medium from macrophages

	TNF-α	IL-6	IL-1β	IL-10	IL-8	MCP-1
CM_GM−_	(5.81 ± 1.11) × 10^−3^	0.15 ± 0.02	N.D.	N.D.	0.10 ± 0.02	1.13 ± 0.19
CM_GM+_	14.16 ± 3.92	11.52 ± 2.85	0.12 ± 0.02	0.16 ± 0.03	5.22 ± 1.57	28.07 ± 4.02
CM_M−_	(5.89 ± 1.32) × 10^−3^	0.12 ± 0.03	N.D.	N.D.	0.03 ± 0.01	1.18 ± 0.37
CM_M+_	2.40 ± 0.40	2.10 ± 0.59	0.03 ± 0.01	2.58 ± 0.48	1.39 ± 0.32	11.05 ± 2.91

Conditioned medium (*CM*) was collected from MΦ_GM_ and MΦ_M_ treated (CM_GM+_ and CM_M+_, respectively) or not (CM_GM−_ and CM_M−_, respectively) with 10 ng/ml LPS for 90 min and incubated 5 h in fresh medium. The data are expressed as ng/ml of culture medium. *N.D.* not detected

### Inflammatory factors secreted by anti-inflammatory macrophages enhance MSC osteogenic potential

**Fig. 3:**
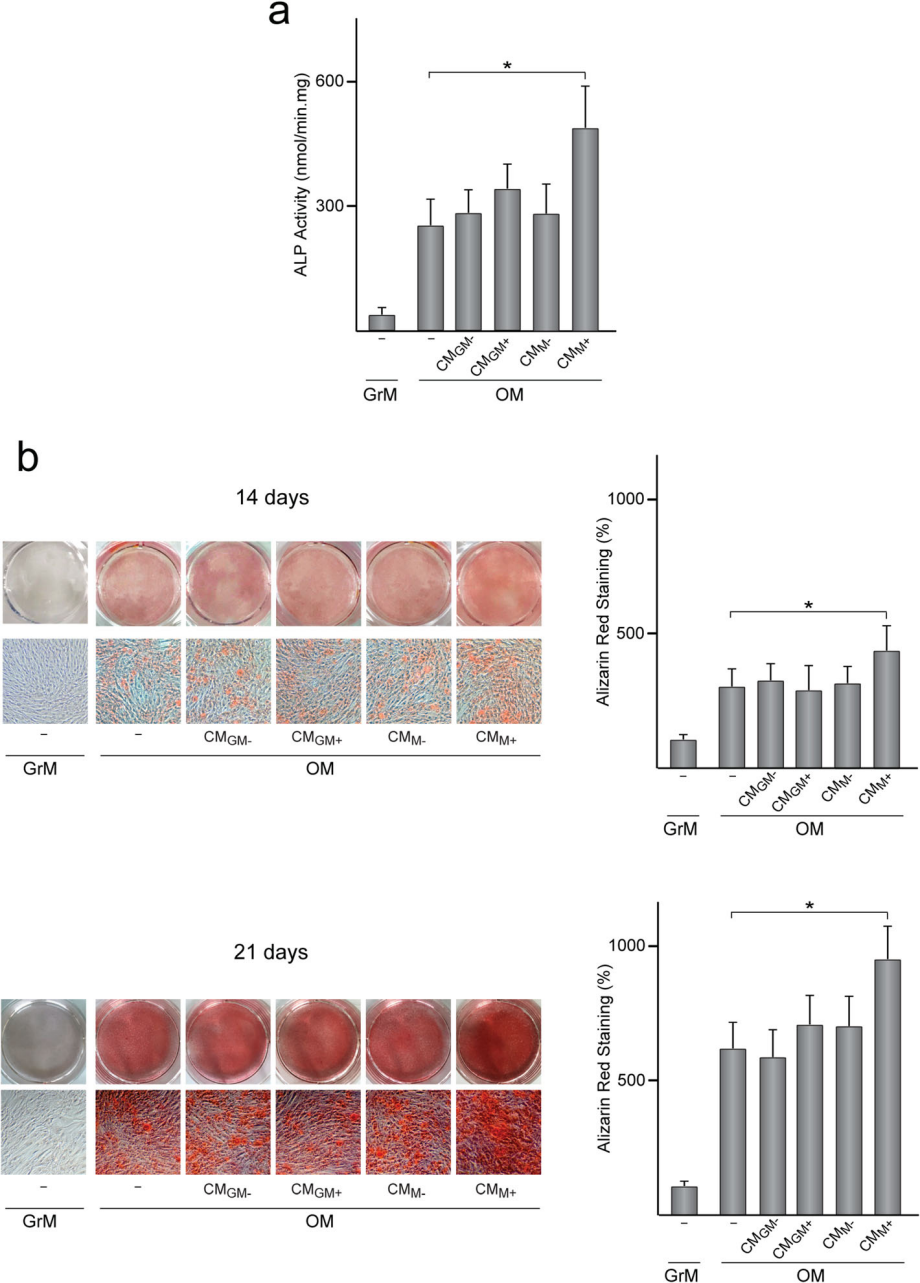
Osteogenic activity of MSC treated with CM. **a** ALP activity in MSC treated or not (−) for 48 h with CM from MΦ_GM_ and MΦ_M_ activated (CM_GM+_ and CM_M+_, respectively) or not (CM_GM−_ and CM_M−_ , respectively) with LPS and further incubated in osteogenic medium (OM) for 14 days. **b.** Alizarin red staining and quantification in MSC treated or not with CM from macrophages and further incubated in OM for 14 days (upper panel) or 21 days (lower panel). Data in b are relative to those measured in untreated MSC incubated in growth medium (GrM), which were given an arbitrary value of 100. *p < 0.05 between the indicated conditions

Next, the effect of inflammatory factors secreted by MΦ_GM_ or MΦ_M_ on MSC osteogenic differentiation was investigated. We performed time-course experiments of ALP activity in MSC treated with CM for 24–96 h and further incubated in osteogenic media for 14 days. Treatment of MSC for 24 h with CM from macrophages, activated or not with LPS, did not lead to significant changes in ALP activity (Additional file 1: Figure S1). ALP activity was neither affected by the treatment of MSC for 48 h with CM from non-activated macrophages or with CM_GM+_ (Fig. 3a). However, MSC treated for 48 h with CM_M+_ showed increased ALP activity as compared to untreated cells (Fig. 3a). Treatment of MSC with CM_M+_ for 72 h also increased ALP activity, although to a lesser extent than treatment for 48 h, whereas treatment for 96 h had no effect (Additional file 1: Figure S1). There was no alizarin red staining indicative of calcium deposits in layers of MSC cultured in osteogenic medium for 3–7 days (Additional file 1: Figure S2). Mineral deposits were visible in MSC layers after 14 days of incubation in osteogenic medium (Fig. 3b). As expected, alizarin red staining was more intense in MSC incubated for 21 than for 14 days (Fig. 3b). At both time points, MSC treated with CM_M+_ showed higher mineralization degree than untreated cells (Fig. 3b).

**Fig. 4:**
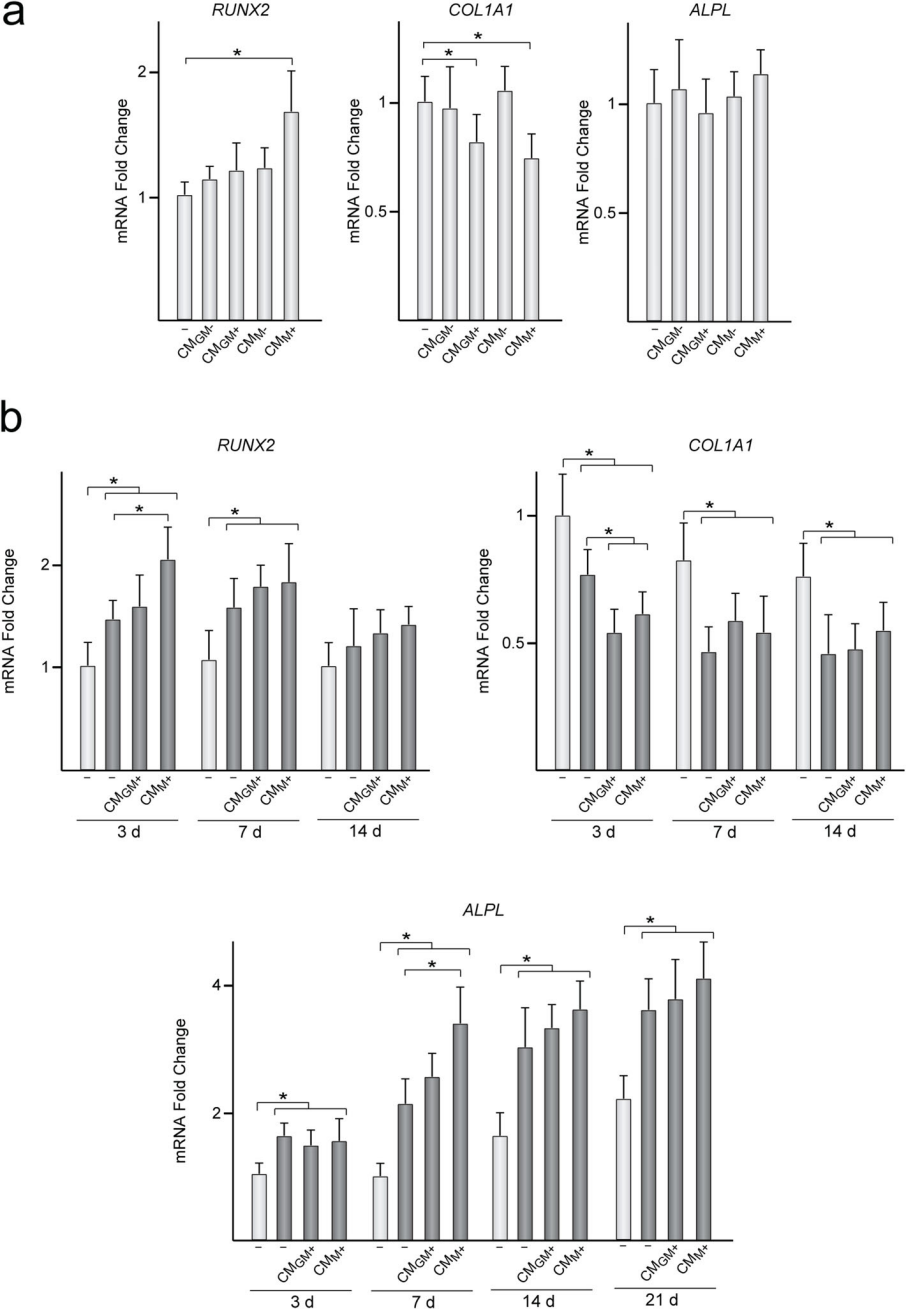
Expression of bone-related genes in MSC treated with CM. **a***RUNX2*, *COL1A1*, and *ALPL* mRNA levels in MSC treated or not (−) for 48 h with CM from MΦ_GM_ and MΦ_M_ activated (CM_GM+_ and CM_M+_, respectively) or not (CM_GM−_ and CM_M−_, respectively) with LPS. Data are relative to those measured in untreated MSC, which were given an arbitrary value of 1. **b***RUNX2*, *COL1A1*, and *ALPL* mRNA levels in MSC treated or not (−) for 48 h with CM_GM+_ or CM_M+_ and further incubated in osteogenic medium (dark gray) for the indicated time points. Data are relative to those measured in untreated MSC incubated in growth medium (light gray) for 3 days, which were given an arbitrary value of 1. *p < 0.05 between the indicated conditions

We then determined whether treatment with inflammatory factors secreted by activated macrophages regulates the expression, at the mRNA level, of osteogenic markers in MSC*.* To this end, mRNA levels of *RUNX2*, *COL1A1*, and *ALPL* were quantified in MSC treated with CM for 48 h. Treatment of MSC with CM from non-activated macrophages did not affect transcript levels of any of the genes tested (Fig. 4a). However, MSC treated with CM_M+_ showed higher *RUNX2* mRNA levels than untreated cells (Fig. 4a). Treating MSC with CM_GM+_ or CM_M+_ led to a decrease in *COL1A1* mRNA levels while *ALPL* mRNA levels remained unaffected (Fig. 4a). Next, we assessed whether treatment with CM modulates the expression of *RUNX2, COL1A1,* and *ALPL* in MSC further incubated in osteogenic medium. In general, the culture of MSC, treated or not with CM, in media with osteogenic inducers increased *RUNX2* and *ALPL* mRNA levels while decreased the levels of *COL1A1* transcripts (Fig. 4b). The expression of these genes in differentiating MSC was not affected by treatment with CM from non-activated macrophages (data not shown). In contrast, *RUNX2* mRNA levels in MSC treated with CM_M+_ were higher than in untreated MSC at 3 days of differentiation. At this time point, *COL1A1* mRNA levels in MSC treated with CM_GM+_ or CM_M+_ were lower than in untreated MSC. When incubation time extended to 7 or 14 days, there were no differences in *RUNX2* or *COL1A1* mRNA levels between MSC untreated and treated with CM (Fig. 4b). Interestingly, after 7 days of incubation in osteogenic medium, MSC treated with CM_M+_ but not with CM_GM+_ showed higher *ALPL* mRNA levels than untreated MSC, whereas no changes were detected thereafter (Fig. 4b). Taking together, these results indicate that the expression of *RUNX2*, *ALPL*, and *COL1A1* in MSC is regulated by factors contained in CM_M+_, an effect associated with increased osteogenic ability.

### TNF-α and IL-10 secreted by anti-inflammatory macrophages are involved in enhanced MSC osteogenesis

**Fig. 5:**
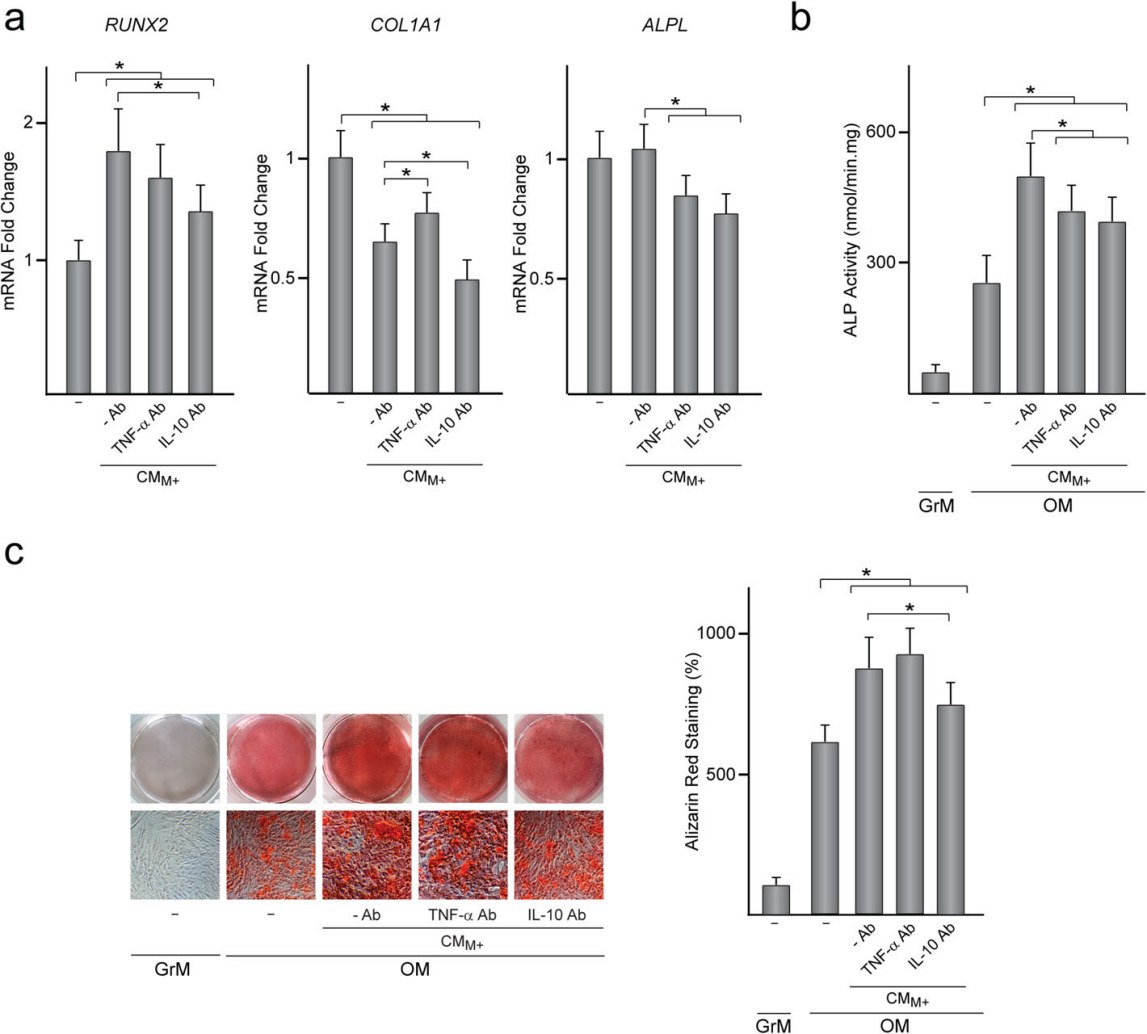
Involvement of TNF-α and IL-10 in the osteogenic activity of MSC treated with CM from anti-inflammatory macrophages. **a***RUNX2*, *COL1A1* and *ALPL* mRNA levels in MSC treated or not (−) for 48 h with CM_M+_ that had been incubated or not (−Ab) with TNF-α or IL-10 neutralizing antibody (Ab). Data are relative to those measured in untreated MSC, which were given an arbitrary value of 1. ALP activity (**b**) and alizarin red staining and quantification (**c**) in MSC treated or not with CM_M+_ and further incubated in osteogenic medium (OM) for 14 (**b**) or 21 (**c**) days. Data in **c** are relative to those measured in untreated MSC incubated in growth medium (GrM), which were given an arbitrary value of 100. *p < 0.05 between the indicated conditions

Treatment of MSC with CM_M+_, which contains higher levels of IL-10 and lower levels of TNF-α than CM_GM+_, enhanced their osteogenic potential. To investigate whether MSC osteogenic differentiation is stimulated by TNF-α and IL-10 secreted by MΦ_M_, MSC were treated for 48 h with CM_M+_ that had been incubated with neutralizing TNF-α or IL-10 antibodies. Blocking IL-10 but not TNF-α attenuated the increase in *RUNX2* mRNA levels induced by treatment with CM_M+_ (Fig. 5a). Notably, *COL1A1* mRNA levels in MSC treated with CM_M+_ increased when TNF-α was blocked whereas neutralization of IL-10 led to the opposite effect (Fig. 5a). *ALPL* mRNA levels in MSC, which remained unaffected by treatment with CM_M+_, were reduced when TNF-α or IL-10 was neutralized (Fig. 5a). Blocking TNF-α or IL-10 led to a decrease in ALP activity of MSC treated with CM_M+_ and further incubated in osteogenic medium (Fig. 5b). In contrast, the formation of mineralized nodules by MSC treated with CM_M+_ decreased by blocking IL-10 but not TNF-α (Fig. 5c). It should be noted that blocking TNF-α or IL-10 in CM_GM+_ had no effect on MSC osteogenesis (Additional file 1: Figure S3). These data indicate that TNF-α and IL-10 secreted by anti-inflammatory macrophages regulate MSC osteogenic activity.

### MSC response to treatment with TNF-α or IL-10

**Fig. 6:**
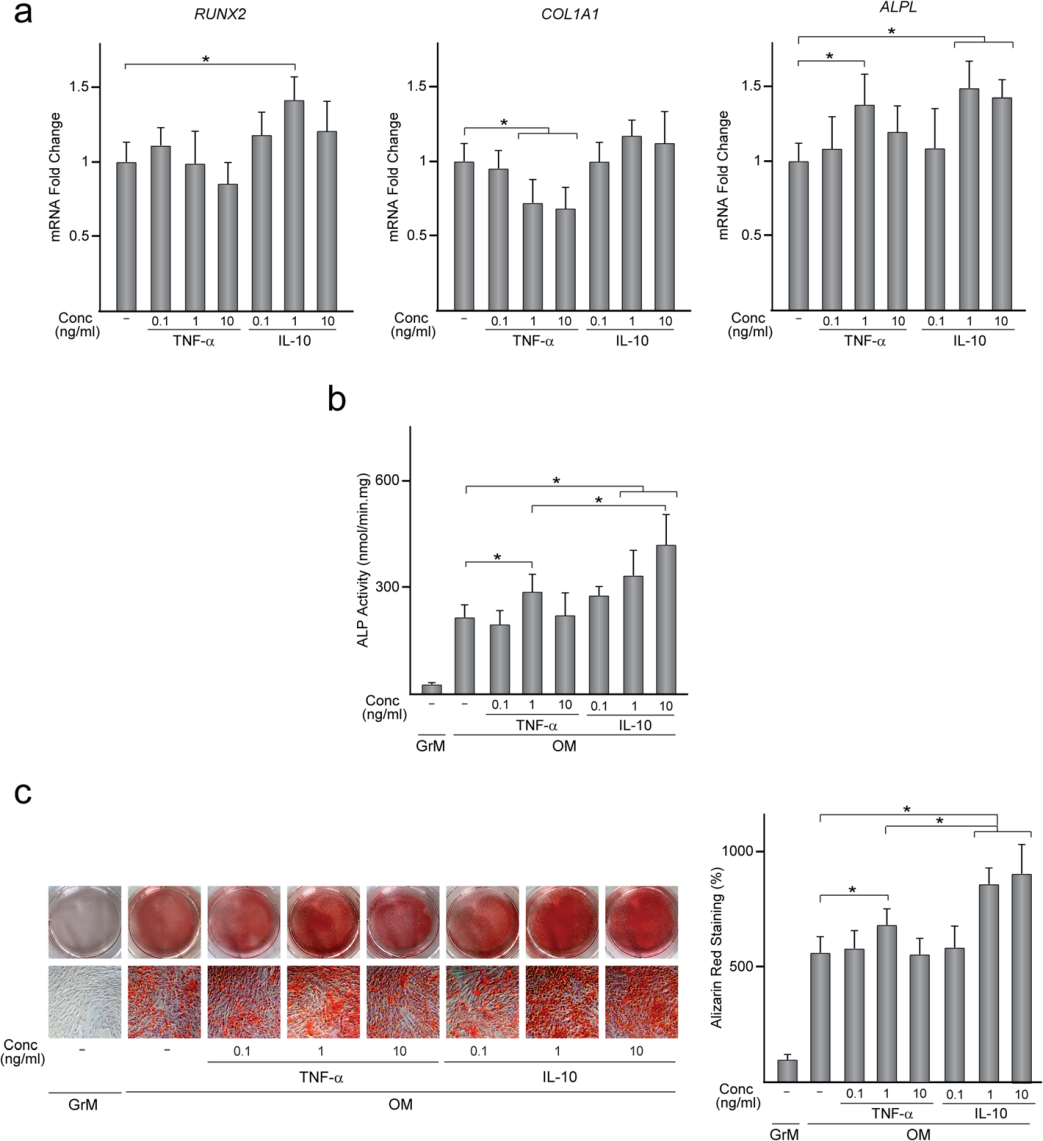
Osteogenic activity of MSC treated with TNF-α or IL-10. **a***RUNX2*, *COL1A1* and *ALPL* mRNA levels in MSC treated or not (−) for 48 h with TNF-α or IL-10 at 0.1, 1, or 10 ng/ml. Data are relative to those measured in untreated MSC, which were given an arbitrary value of 1. ALP activity (**b**) and alizarin red staining and quantification (**c**) in MSC treated or not with TNF-α or IL-10 and further incubated in osteogenic medium (OM) for 14 (**b**) or 21 (**c**) days. Data in **c** are relative to those measured in untreated MSC incubated in growth medium (GrM), which were given an arbitrary value of 100. *p < 0.05 between the indicated conditions

**Fig. 7:**
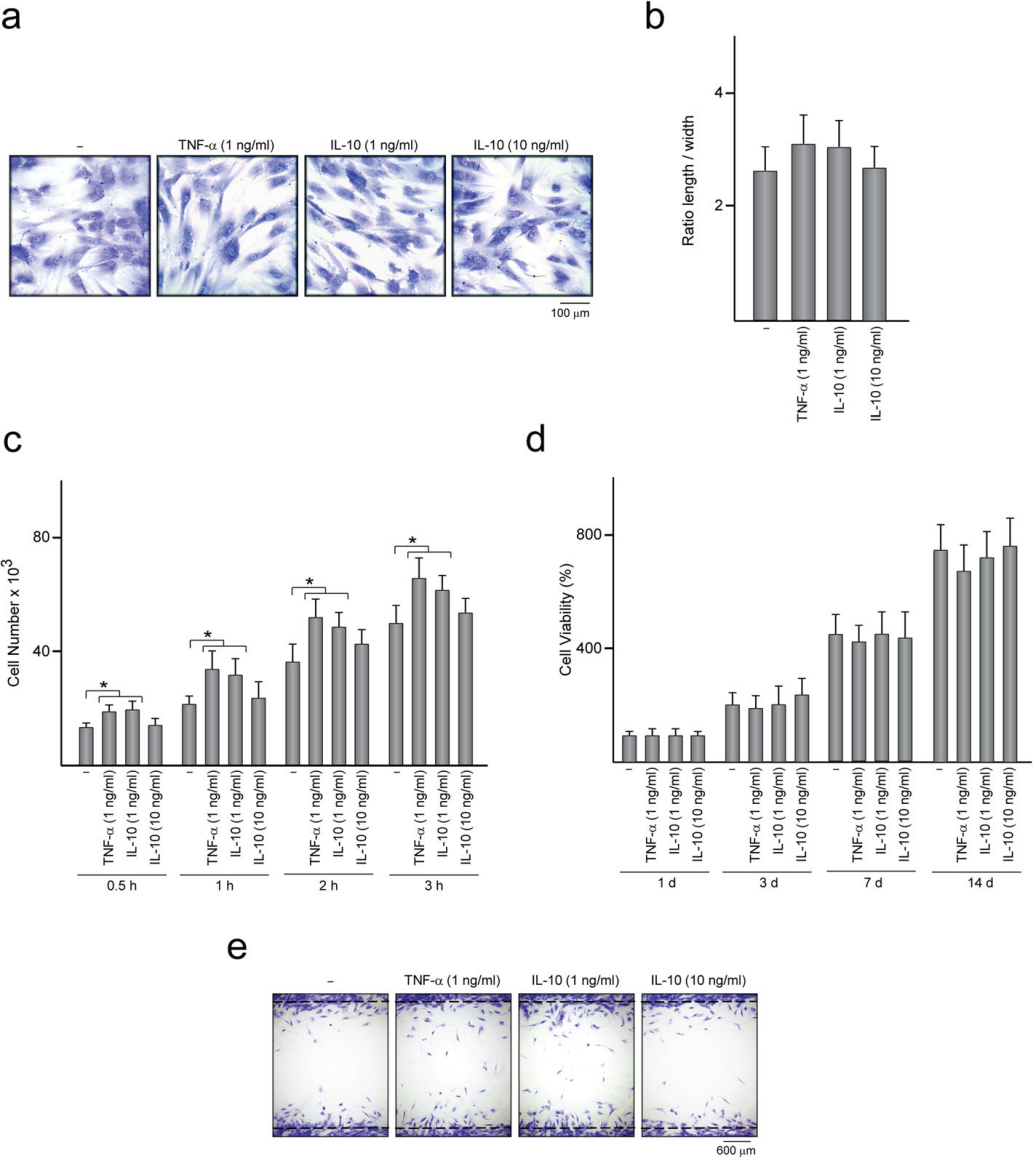
Morphology, adhesion, viability, and migration of MSC treated with TNF-α or IL-10. **a** Optical microscope images showing the morphology of MSC treated or not (−) for 48 h with the indicated doses of TNF-α or IL-10, after crystal violet staining. **b** Ratio of cell length to width as a measure of cell elongation. **c** Number of MSC treated or not with TNF-α or IL-10 and incubated in growth medium (GrM) for the indicated time points. **d** Viability of MSC treated or not with TNF-α or IL-10 and further incubated in GrM for the indicated time points. The data are relative to those measured in each group on day 1, which were given an arbitrary value of 100. **e** Migration capacity of MSC seeded inside the area confined by squared silicone barriers, treated or not with TNF-α or IL-10 and allowed to migrate for 5 days. Optical microscope images of cells stained with crystal violet. *p < 0.05 between the indicated conditions

To further investigate the effect of TNF-α and IL-10 on MSC osteogenesis, MSC were treated for 48 h with 0.1, 1, or 10 ng/ml TNF-α or IL-10 and mRNA levels of *RUNX2*, *COL1A1*, and *ALPL* were quantified (Fig. 6a). *RUNX2* transcript levels increased only when MSC were treated with 1 ng/ml IL-10. *COL1A1* mRNA levels decreased after treating MSC with TNF-α at 1 or 10 ng/ml while IL-10 had no effect at any tested dose. The effects of IL-10 or TNF-α on *ALPL* expression were dependent on the assayed dose. *ALPL* mRNA levels in MSC remained unaffected by treatment with IL-10 at the lowest dose while increased when IL-10 was applied at 1 or 10 ng/ml. TNF-α at 1 ng/ml also increased *ALPL* mRNA levels in MSC whereas it had no effect at 0.1 or 10 ng/ml. Treatment with TNF-α or IL-10 at 1 ng/ml increased ALP activity of MSC incubated under osteogenic conditions, which further increased by IL-10 at 10 ng/ml (Fig. 6b). In addition, mineralized nodule formation in MSC layers increased after treatment with 1 ng/ml TNF-α and further increased with IL-10 at 1 or 10 ng/ml (Fig. 6c). Treatment of MSC with IL-10 or TNF-α at the lowest dose or with 10 ng/ml TNF-α had no effect on MSC osteogenic differentiation (Fig. 6b, c). As incubation of MSC with CM from macrophages enhances MSC adhesion and migration (Fig. 2a, c), we finally evaluated whether TNF-α and IL-10, at doses that enhance osteogenesis, also modulate these processes. MSC treated with cytokines displayed an elongated or irregularly polygonal shape, similar to that observed in untreated cells (Fig. 7a). In fact, the average elongation ratio of MSC untreated and treated with cytokines was similar (Fig. 7b). MSC attachment was unaffected by treatment with IL-10 at 10 ng/ml (Fig. 7c). In contrast, MSC treated with TNF-α or IL-10 at 1 ng/ml attached at a higher extent than untreated MSC (Fig. 7c). Treatment with TNF-α and IL-10 did not affect MSC viability (Fig. 7d). As observed for cell attachment, MSC migration increased upon treatment with TNF-α or IL-10 at 1 ng/ml (Fig. 7e). Together, these data indicate that treatment with TNF-α and IL-10 at 1 ng/ml enhances MSC adhesion, migration and osteogenesis.

## Discussion

Macrophages play a key role during bone healing, participating in both initial inflammatory response and early anabolic phase of repair [1]. Phenotypic switching of macrophages during fracture healing leads to temporally controlled release of inflammatory mediators [26]. While the prominent contribution of inflammatory factors to fracture healing is well established, their direct effects on osteogenic precursors are not fully understood. Having recently shown that factors originated from pro-inflammatory and anti-inflammatory macrophages enhance MSC immunoregulatory properties [18], data herein provide evidence that MSC adhesion, migration, and osteogenic potential are also regulated by the inflammatory environment. Inflammatory cytokines influence focal adhesion assembly and cytoskeleton rearrangement, which regulate cell adhesion and migration [27, 28]. MSC treated with CM from activated macrophages elongated as a result of a more parallel arrangement of actin bundles. Cell morphological elongation has been associated with an increased migration rate [29]. In this regard, migratory activity was higher in MSC treated with CM_GM+_ or CM_M+_ than with CM from non-activated macrophages, supporting the involvement of inflammatory cytokines in MSC mobilization. In fact, treatment with TNF-α or IL-10 enhanced MSC ability to attach and migrate, although to a lesser extent than CM from activated macrophages. Of note, CM_GM+_ increased MSC elongation, attachment, and migration to a higher extent than CM_M+_, suggesting that pro-inflammatory factors that participate in the early inflammatory response enhance MSC mobilization to injured sites. It should be mentioned that no obvious differences in cell elongation were noted between differentiating MSC treated with CM and untreated MSC (Additional file 1: Figure S2), indicating that the effect of CM on cell morphology was rapidly lost in osteogenic medium.

The role of macrophage-derived factors in MSC osteogenesis remains controversial, mainly due to differences in experimental setups used to study it [30]. Previous studies have reported that the treatment of MSC with media conditioned by pro-inflammatory macrophages increases *RUNX2* and *ALPL* mRNA levels [31] as well as cell layer mineralization [32]. However, we found that the treatment of MSC with CM_GM+_ did not result in increased MSC osteogenic differentiation. A plausible explanation for this discrepancy is that MSC osteogenesis was conducted in our experiments in the absence of LPS whereas the previous studies used CM containing LPS as stimulus. LPS regulates MSC osteogenic differentiation, as treatment of MSC with TNF-α plus LPS, but not with TNF-α only, enhanced ALP activity and cell layer mineralization [22]. Unlike M1-like phenotype, murine bone marrow macrophages [33] and THP-1 cells [34] polarized to an M2-like phenotype release factors that enhance MSC osteogenic differentiation, as observed in co-cultures that allow the exchange of soluble factors without direct cell contact. In line with these data, we observed enhanced ALP activity in MSC treated with factors secreted by human anti-inflammatory macrophages at 14 days of differentiation. Enhanced mineralized nodule formation was also detected at this time, indicating earlier calcium deposition to the matrix in MSC after exposure to CM_M+_. Therefore, factors secreted by pro-resolving macrophages during the resolution of inflammation might contribute to create a more favorable environment for MSC osteogenesis than those released by pro-inflammatory macrophages at the early stage of inflammation.

Inflammatory cytokines secreted by macrophages contribute to bone formation, as injection of low-doses of TNF-α at the fracture site accelerated bone healing in mice [19]. Investigations into the role of TNF-α on osteogenic differentiation of stromal cells have been mainly conducted using recombinant proteins. In the present study, we show that TNF-α produced by macrophages regulates MSC osteogenic potential. Culture of MSC with CM_M+_ incubated with a blocking anti-TNF-α antibody attenuated the initial phase of osteogenesis, as ALP induction was partially inhibited, whereas blocking TNF-α in CM_GM+_ did not affect MSC osteogenic ability. These data suggest that TNF-α may exert osteogenic effects at the onset of the resolution, when its levels start to decrease. Interestingly, exposure of MSC to 1 ng/ml TNF-α increased ALP activity whereas 10 ng/ml TNF-α had no effect on MSC osteogenic differentiation. It is worth mentioning that 1 ng/ml TNF-α is actually close to the mean concentration, of about 700 pg/ml, detected in serum samples from fracture hematomas of patients within 72 h post-injury [35].

The influence of pro-resolving mediators on MSC osteogenesis remains poorly understood. Phenotypic switching of macrophages during fracture repair leads to the production of IL-10, found in serum samples from fracture hematomas of patients at concentrations around 1 ng/ml [36]. A recent study showed that recombinant IL-10 can enhance MSC osteogenesis via the p38/mitogen-activated protein kinase (MAPK) signaling pathway [25]. To our knowledge, our study is the first to demonstrate that IL-10 secreted by macrophages regulates MSC osteogenic differentiation. CM_GM+_ contained very low levels of IL-10, which had no effect on MSC osteogenesis. However, significant IL-10 levels secreted by anti-inflammatory macrophages enhanced MSC osteogenic ability, as blocking IL-10 led to a reduction in ALP activity and mineralization in MSC treated with CM_M+_. Experiments in which MSC were treated with recombinant cytokines revealed that IL-10 was indeed more effective in promoting matrix mineralization than TNF-α. In addition to promote MSC osteogenesis, IL-10 enhanced MSC attachment and migration at a dose of 1 ng/ml but not at 10 ng/ml, suggesting that MSC functions could be tightly regulated by anti-inflammatory mediators in the resolution of inflammation. It should be noted that the effects of TNF-α and IL-10 on in vitro MSC osteogenesis likely depend on the length of their application, being pro-osteogenic after short-term treatment, as in the present study, or inhibitory after continuous exposure [23, 25]. Based on our observations, we speculate that the controlled release of inflammatory signals by macrophages after injury could promote MSC osteogenesis. However, exacerbated and/or sustained pro-inflammatory response could lead to chronic inflammation and tissue destruction whereas excessive anti-inflammatory macrophage activation could promote fibrosis and abnormal extracellular matrix deposition.

Our data support the involvement of RUNX2 in early stages of osteogenesis, as incubation of MSC in osteogenic medium for 3 days increased *RUNX2* mRNA levels. A recent study showed that, in the absence of classic osteogenic inducers, treatment of mouse mesenchymal precursors with cytokines like IL-13 or IL-17A increased the expression of RUNX2, at the protein level, and induced osteoblast differentiation [37]. Our data support the role of cytokines in regulating RUNX2, as treatment of MSC with CM_M+_ for 48 h increased *RUNX2* mRNA levels, an effect partially mediated by IL-10. MSC treated with recombinant IL-10 at 1 ng/ml showed higher *RUNX2* mRNA levels than untreated cells, supporting the role of IL-10 in promoting MSC osteogenesis. No significant changes in *ALPL* expression were observed in MSC immediately after treatment with CM_M+_. However, exposure to factors released by anti-inflammatory macrophages enhanced *ALPL* expression in early differentiating MSC, as MSC treated with CM_M+_ and incubated in osteogenic media for 7 days showed higher *ALPL* mRNA levels than untreated cells. *COL1A1* is related to the proliferation period and its expression decreases when differentiation starts. mRNA levels of *COL1A1*, which contains *RUNX2* binding sites in its promoter [38], decreased after treating MSC with CM_M+_, suggesting further layers of transcriptional regulation. In this regard, TNF-α is known to reduce collagen type I expression [39]. In fact, the culture of MSC with CM_M+_ incubated with anti-TNF-α increased *COL1A1* mRNA levels and treatment of MSC with TNF-α at 1 or 10 ng/ml decreased *COL1A1* expression. Interestingly, *COL1A1* mRNA levels in MSC treated with CM_M+_ decreased when IL-10 was blocked, indicating that IL-10 and TNF-α in CM_M+_ have opposite effects on *COL1A1* expression. These results suggest that a correct balance between pro- and anti-inflammatory cytokines is necessary for successful collagen matrix deposition after bone injury. Of note, treatment of MSC with IL-10 only did not alter *COL1A1* mRNA levels at any tested dose, which indicated that other factors secreted by anti-inflammatory macrophages cooperate with IL-10 in the regulation of *COL1A1* expression.

In summary, data in this study support the hypothesis that inflammatory environment promotes MSC osteogenesis at the onset of resolution rather than at the early stage of inflammation, which is more favorable for MSC recruitment. We have demonstrated that IL-10 secreted by anti-inflammatory macrophages potentiates MSC osteogenesis by increasing the initial phase of matrix maturation as well as mineralization. In addition, IL-10 enhances MSC ability to attach and migrate, which in turn may contribute to bone formation. It should be noted that, in addition to TNF-α and IL-10, CM may contain a large range of soluble factors that could contribute to regulate MSC osteogenesis. Current efforts to augment bone repair involve the use of cytokines for local administration at the fracture site or for priming protocols before MSC implantation [40, 41]. Data herein provide new insights into how cytokines in different inflammatory environments modulate MSC osteogenesis, which may be helpful to develop effective bone regeneration strategies.

## Conclusions

Factors secreted by pro-inflammatory or anti-inflammatory macrophages increased MSC attachment and migration. These effects were more pronounced when MSC were treated with media from pro-inflammatory macrophages. MSC osteogenic activity was enhanced by factors released by anti-inflammatory macrophages, but not by pro-inflammatory macrophages. We found that IL-10 originated from anti-inflammatory macrophages increased MSC osteogenic differentiation and regulated the expression of osteogenic markers. In addition, IL-10 potentiated the MSC ability to attach and migrate. These findings contribute to the understanding of the mechanisms by which phenotypic switching of macrophages during bone repair influences MSC osteogenesis and migration.

## Supplementary information

**Additional file 1: Figure S1.** ALP activity in MSC treated for 24, 72 or 96 h with CM from MΦGM and MΦM activated (CMGM+ and CMM+, respectively) or not (CMGM- and CMM-, respectively) with LPS and further incubated in osteogenic medium (OM) for 14 days. Untreated MSC (−) were incubated in growth medium (GrM) or OM for 14 days. *p < 0.05 between the indicated conditions. **Figure S2.** Alizarin Red S staining in MSC treated with CM from MΦGM and MΦM activated (CMGM+ and CMM+, respectively) or not (CMGM- and CMM-, respectively) with LPS and further incubated in osteogenic medium (OM) for 3 or 7 days. Untreated MSC (−) were incubated in growth medium (GrM) or OM for 3 or 7 days. **Figure S3. **Involvement of TNF-α and IL-10 in the osteogenic activity of MSC treated with CM from pro-inflammatory macrophages. MSC were treated or not (−) for 48 h with CMGM+ that had been incubated or not (−Ab) with TNF-α or IL-10 neutralizing antibody (Ab). ALP activity (a) and alizarin Red S staining and quantification (b) in MSC treated or not with CMGM+ and further incubated in OM for 14 (a) or 21 (b) days. Data in b are relative to those measured in untreated MSC incubated in growth medium (GrM), which were given an arbitrary value of 100.

## Data Availability

The datasets generated and/or analyzed during the current study are available from the corresponding author on reasonable request.

## Abbreviations

ALP: Alkaline phosphatase
CM: Conditioned medium
CM_GM−_: CM from non-activated MΦ_GM_
CM_GM+_: CM from LPS-activated MΦ_GM_
CM_M−_: CM from non-activated MΦ_M_
CM_M+_: CM from LPS-activated MΦ_M_
COL1A1: Collagen type I alpha 1
FBS: Fetal bovine serum
GM-CSF: Granulocyte macrophage colony-stimulating factor
IL: Interleukin
LPS: Lipopolysaccharide
MCP-1: Monocyte chemoattractant protein-1
M-CSF: Macrophage colony-stimulating factor
MSC: Mesenchymal stem cells
MΦ_GM_: GM-CSF-generated macrophages
MΦ_M_: M-CSF-generated macrophages
PBMC: Peripheral blood mononuclear cells
PBS: Phosphate-buffered saline
RUNX2: Runt-related transcription factor 2
TNF-α: tumor necrosis factor-alpha
