# Supplementary material for: Influence of inflammatory conditions provided by macrophages on osteogenic ability of mesenchymal stem cells
Source: Stem Cell Res Ther. 2020 Feb 13;11:57. doi: 10.1186/s13287-020-1578-1 (PMC7020593; doi:10.1186/s13287-020-1578-1)
Supplement: Supplementary file 1 — Additional file 1: Figure S1. ALP activity in MSC treated for 24, 72 or 96 h with CM from MΦGM and MΦM activated (CMGM+ and CMM+, respectively) or not (CMGM- and CMM-, respectively) with LPS and further incubated in osteogenic medium (OM) for 14 days. Untreated MSC (−) were incubated in growth medium (GrM) or OM for 14 days. *p < 0.05 between the indicated conditions. Figure S2. Alizarin Red S staining in MSC treated with CM from MΦGM and MΦM activated (CMGM+ and CMM+, respectively) or not (CMGM- and CMM-, respectively) with LPS and further incubated in osteogenic medium (OM) for 3 or 7 days. Untreated MSC (−) were incubated in growth medium (GrM) or OM for 3 or 7 days. Figure S3. Involvement of TNF-α and IL-10 in the osteogenic activity of MSC treated with CM from pro-inflammatory macrophages. MSC were treated or not (−) for 48 h with CMGM+ that had been incubated or not (−Ab) with TNF-α or IL-10 neutralizing antibody (Ab). ALP activity (a) and alizarin Red S staining and quantification (b) in MSC treated or not with CMGM+ and further incubated in OM for 14 (a) or 21 (b) days. Data in b are relative to those measured in untreated MSC incubated in growth medium (GrM), which were given an arbitrary value of 100. [file 13287_2020_1578_MOESM1_ESM.pdf]

Additional File 1

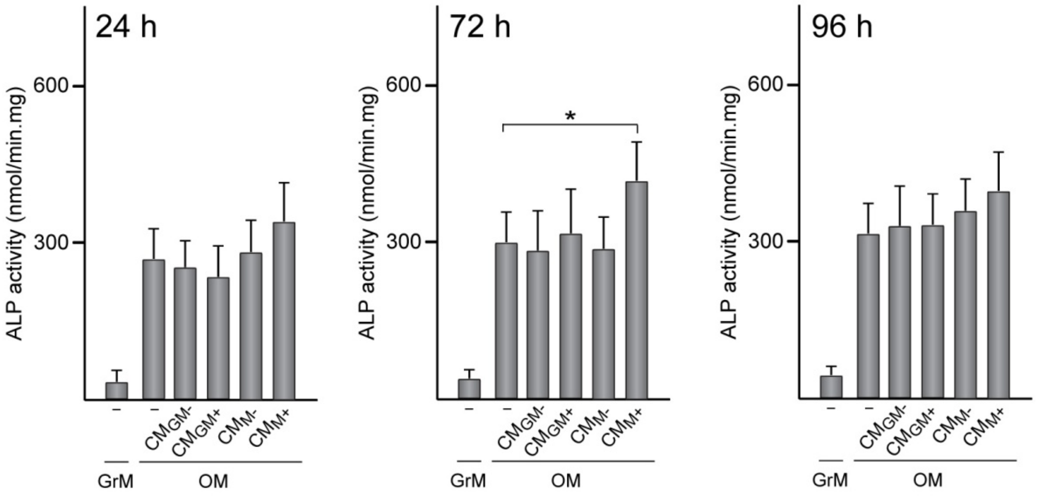

Figure S1. ALP activity in MSC treated for 24, 72 or 96 h with CM from $M\Phi_{GM}$ and $M\Phi_{M}$ activated (CM_{GM+} and CM_{M+}, respectively) or not (CM_{GM-} and CM_{M-}, respectively) with LPS and further incubated in osteogenic medium (OM) for 14 days. Untreated MSC (-) were incubated in growth medium (GrM) or OM for 14 days. $*p < 0.05$ between the indicated conditions.

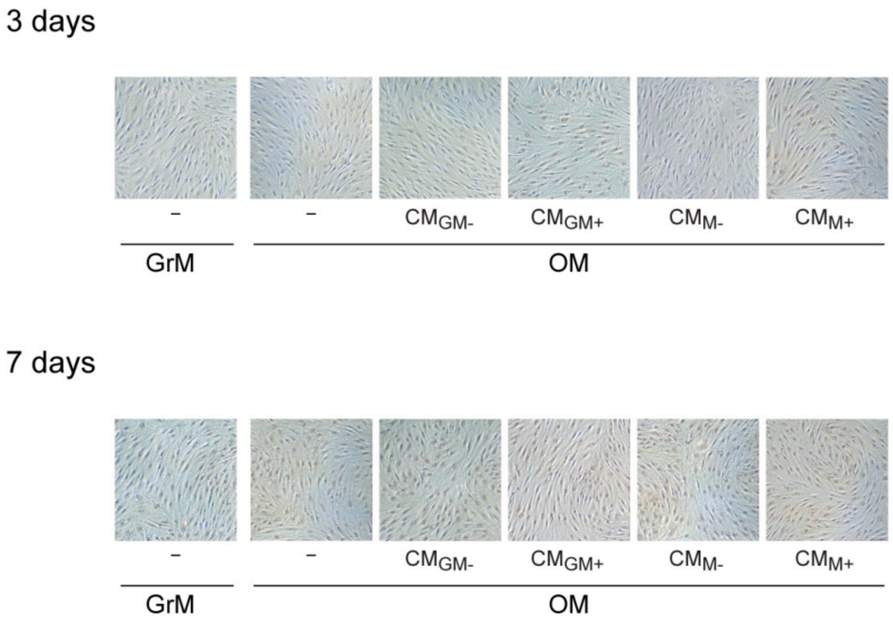

Figure S2. Alizarin Red S staining in MSC treated with CM from $M\Phi_{GM}$ and $M\Phi_{M}$ activated (CM_{GM+} and CM_{M+}, respectively) or not (CM_{GM-} and CM_{M-}, respectively) with LPS and further incubated in osteogenic medium (OM) for 3 or 7 days. Untreated MSC (-) were incubated in growth medium (GrM) or OM for 3 or 7 days.

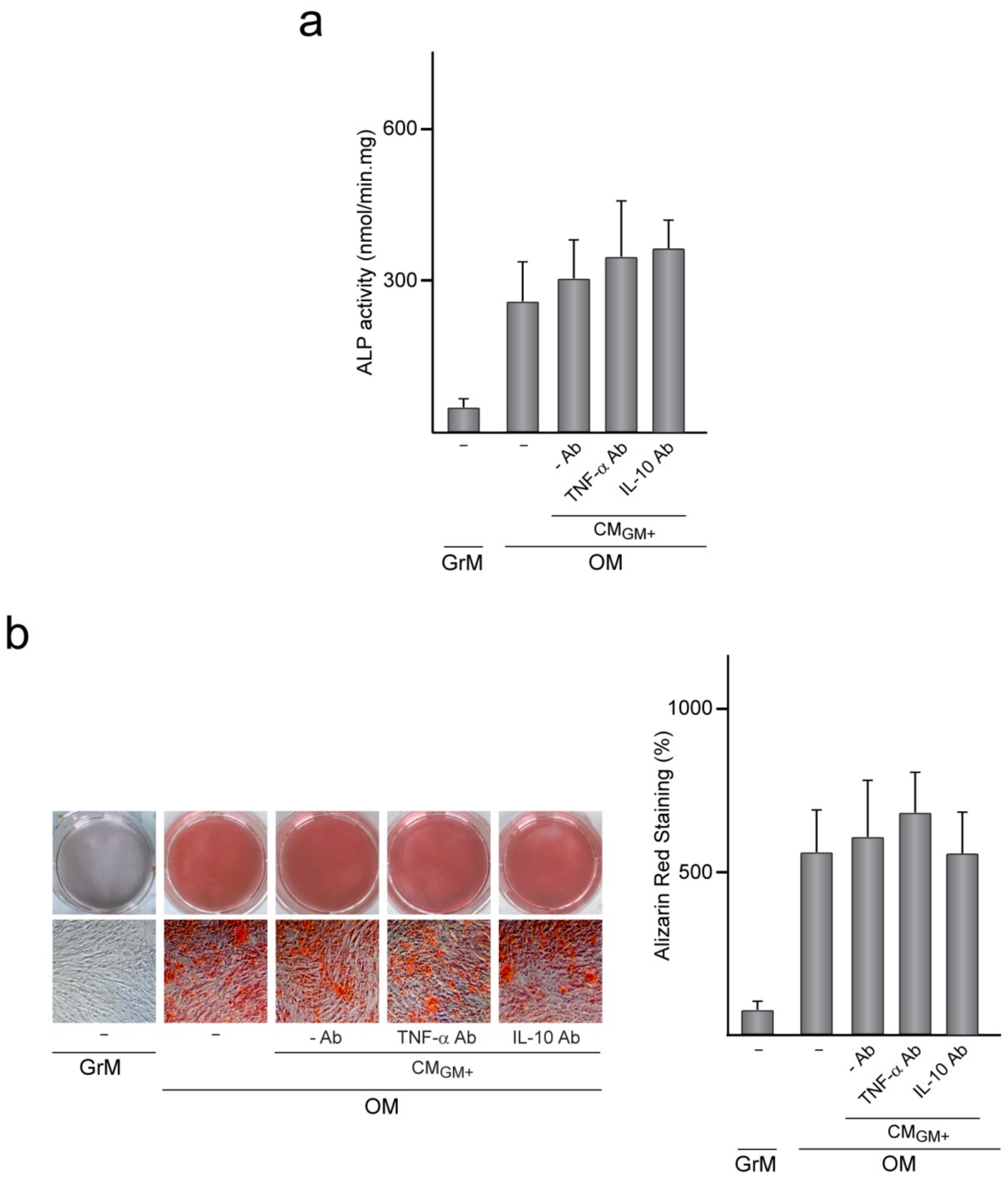

Figure S3. Involvement of TNF-α and IL-10 in the osteogenic activity of MSC treated with CM from pro-inflammatory macrophages. MSC were treated or not (-) for 48 h with CM_{GM+} that had been incubated or not (-Ab) with TNF-α or IL-10 neutralizing antibody (Ab). ALP activity (a) and alizarin Red S staining and quantification (b) in MSC treated or not with CM_{GM+} and further incubated in OM for 14 (a) or 21 (b) days. Data in b are relative to those measured in untreated MSC incubated in growth medium (GrM), which were given an arbitrary value of 100.
